# Bullous striae distensae in a nephrotic syndrome patient: First case report from the Middle East of a rare presentation and review of the literature

**DOI:** 10.1002/ccr3.8667

**Published:** 2024-04-05

**Authors:** Sumayyah I. Alrefaie, Sarah B. Aljoudi, Houriah Y. Nukaly, Waseem K. ALHawasawi, Jehad O. Hariri

**Affiliations:** ^1^ Faculty of Medicine King Abdulaziz University Jeddah Saudi Arabia; ^2^ Department of Dermatology, Faculty of Medicine King Abdulaziz University Jeddah Saudi Arabia; ^3^ College of Medicine and Surgery Batterjee Medical College Jeddah Saudi Arabia; ^4^ Department of Dermatology King Fahad Armed Forces Hospital Jeddah Saudi Arabia

**Keywords:** bullae, corticosteroid, edema, nephrotic syndrome, striae distensa

## Abstract

Striae distensae is a common cutaneous phenomenon that begins as reddish linear atrophic plaques (striae rubra) that over time progress to silvery‐white coloration (stria alba). Striae distensae in rare occasions becomes edematous, ulcerative, emphysematous, or urticated. Bullous striae distensae is a sequela of conditions causing interstitial edema along with systemic glucocorticoids use. To our knowledge, only eight cases of bullous striae distensae have been reported in the literature. Herein, we report a 17‐year‐old female, known case of nephrotic syndrome, presented to our clinic with abdominal fluid‐fill cutaneous lesions only for 5 days. She had used systemic glucocorticoids for more than a decade before she was labeled as steroid resistant nephrotic syndrome. Cushingoid body habitus were observed during physical examination, in addition to translucent bullae overlying her previously known stretch marks. Punch biopsy of the lesions revealed dermal edema with thinned collagen bundles. Based on these clinicopathological findings, a diagnosis of bullous striae distensae was made. Awareness of this rare complication and unusual clinical presentation is fundamental to avoid unnecessary and excessive interventions whether investigatory or therapeutic in order to provide appropriate management of the underlying condition.

## 1 INTRODUCTION

Striae distensae (SD), a well‐recognized phenomenon resulting from dermal scaring, it is usually associated with physiological conditions such as pregnancy, growth spurt, rapid weight loss or gain or iatrogenic causes such as topical or systemic corticosteroid administration.^1^ It is aesthetically troublesome and therapeutically challenging. SD early stages appear as flesh‐toned atrophic linear plaques that eventually progress to silvery‐whitish atrophic plaques.^1^, ^2^ Most commonly affect dispensable body areas such as the buttock, lower back, thighs, calves, breast and abdomen.^3^ Rare secondary changes within SD have been mentioned in the literature, including edema, urticaria, dyspigmentation, ulceration, dehiscence, and subcutaneous emphysema. Fluid filling these striae are an unusual finding. Very few cases have been reported discussing this phenomenon.^4^, ^5^ Upon literature review, it was notable that even in the very few case reports of BSD, almost all the patients were on long‐term oral steroids as well as having hypoalbuminemia, except for one patient as demonstrated in Table 1.^4^ Herein, we report a case of a 17‐year‐old girl with nephrotic syndrome who was treated with high dose of corticosteroids presented to the day‐care unit with bullous striae distensae.

**TABLE 1:** Main findings from literature review addressing each of the selected domains.

Title	Authors	Age (years), gender	Comorbidities	Cutaneous findings	Investigation findings	Management plan
Bullous striae distensae	Vishal Gupta et al. (2016)^2^	17, female	Nephrotic syndrome	Multiple striae on the axillae, abdomen, lower back, buttocks, and thighs. The striae on the flanks and lower abdomen appeared to be swollen and shiny, and clear serous fluid came out from them on puncturing with a sterile needle	Proteinuria +4 Anemia Hypoalbuminemia	Not mentioned
Fluid within striae—An Unusual Presentation	Divya Seshadri et al. (2013)^3^	14, female	Nephrotic syndrome	Swollen, bulged out shiny, translucent abdominal striae	High urine albumin Hypoalbuminemia	Not mentioned
Fluid‐filled striae in a patient with hypoalbuminemia	Maria Jogova et al. (2017)^4^	57, female	Protein losing enteropathy secondary to C. Difficile infection	Numerous linear, lobulated, fluid‐filled skin lesions that flattened with transient pressure on the limbs and trunk	Hypoalbuminemia High urine protein	Antibiotics Diuresis
A Case of edematous striae distensae in lupus nephritis	Lee JH et al. (1998)^5^	17, female	SLE (lupus nephritis)	Painful edematous striae distensae on the abdomen.	Speckled ANA Positive anti‐dsDNA Positive SSA antigen Positive ENA antigen Low serum C3 levels Proteinuria +3 Hypoalbuminemia	400 mg/day of furosemide 62.5 mg/day of systemic methylprednisolone
Bullous striae distensae with prolonged steroid use: An unusual clinical presentation	Sadia Masood et al. (2020)^6^	28, female	Pregnant with SLE	Abdominal and breast striae with fluids	Positive ANA Positive anti‐dsDNA	400 mg hydroxychloroquine along with SLE medications
Infected Edematous Striae Distensae	Salma Ahmad Almashat et al. (2021)^7^	13, female	Nephrotic syndrome	Diffuse abdominal redness and distention with fluid‐filled striae	High White Blood cell count (WBC) High D‐dimer Hypoalbuminemia High urine protein	Amoxicillin 1 gram (bis in die) BID; Twice a day.
Yellow bullous striae distensae	Chaocheng Liu et al. (2022)^8^	25, male	Dermatomyositis with liver dysfunction	Yellow linear bullous eruption over his inner arms	Hypoalbuminemia Elevated liver function tests (LFTs)	Not Mentioned
Bullous Striae Distensae: First Case Report in The Middle East of a Rare Presentation (current case)	Alrefaie et al. (2023)	17, female	Nephrotic syndrome	Multiple translucent bullae along the striae distribution on her abdomen and extending to the flanks	Hypoproteinaemia Abnormal lipid profile Anemia Neutrophilic leucocytosis Lymphopenia Thrombocytosis Hypocalcaemia High microalbumin/creatinine ratio Abnormal urine analysis Fluid aspiration: ‐Fluid albumin level was 0.3 ‐WBC count was 42 cells\mm^3^	Diuresis

Abbreviations: ANA, antinuclear antibody test; Anti‐dsDNA, anti‐double stranded DNA antibodies; C3 Levels, complement (C3) levels; ENA antigen, extractable nuclear antigen; SSA antigen, Sjögren's‐syndrome‐related antigen.

## 2 LITERATURE REVIEW

Table 1 Main findings from literature review addressing each of the selected domains.

## 3 CASE HISTORY

We present a 17‐year‐old female who was referred to us from the day care unit for the management of bullous lesions that started to appear on her abdomen 5 days prior. The patient was known to have nephrotic syndrome since the age of 1 year. Initially she had steroid dependent nephrotic syndrome for years and had received fluctuating doses of systemic steroids for a long duration. Later, she was diagnosed with steroid resistant nephrotic syndrome at the age of 12.

**FIGURE 1:**
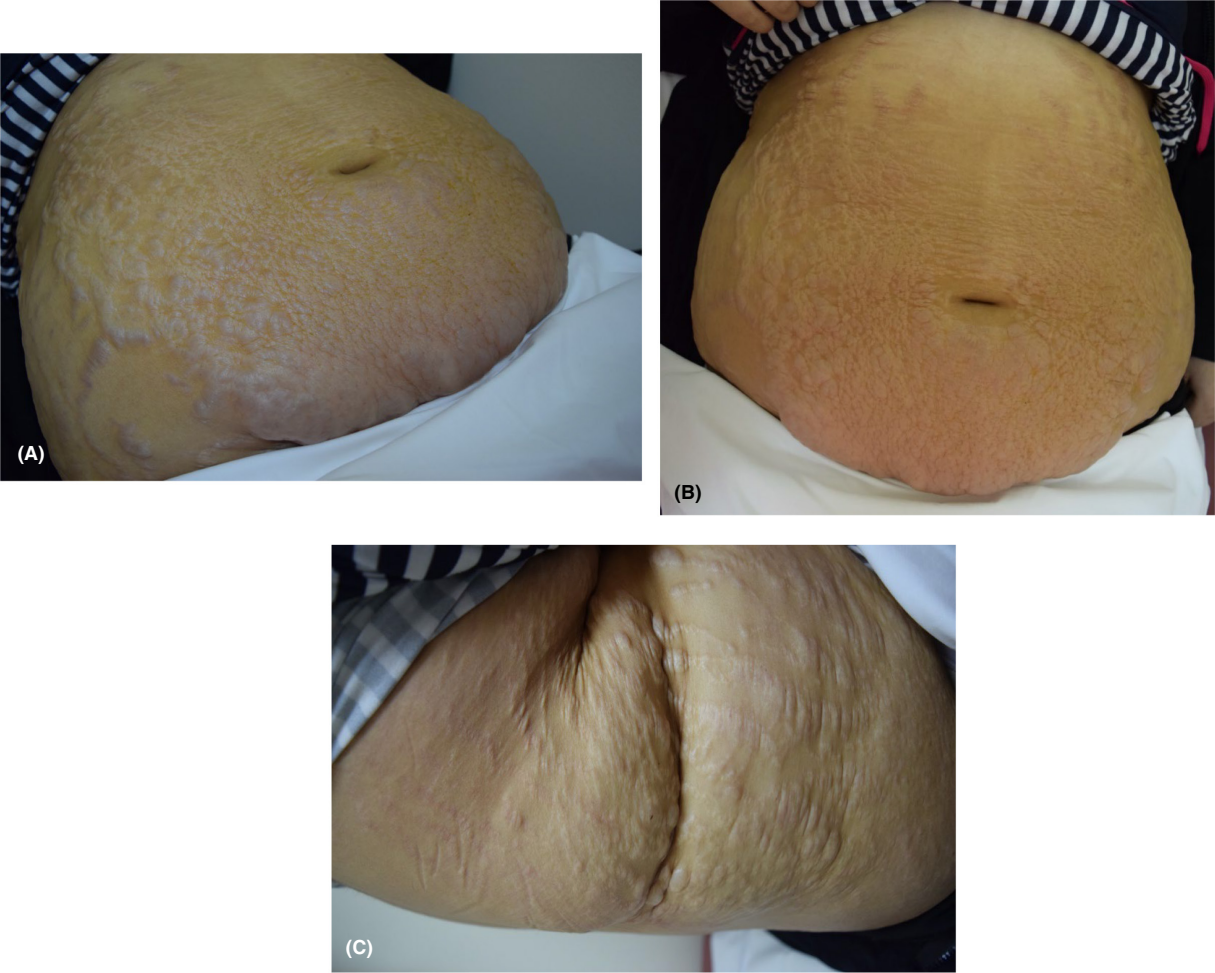
Bullous striae distensae. Multiple transcluent bullae along the striae distribution on the (A and B) abdomen and (C) the flanks in a 17 years old female with nephrotic syndrome.

Upon physical examination, she was found to have a cushingoid body habitus, moon face, central obesity, abdominal striae, and ascites. Rapid weight gain was also noticed recently. There were multiple translucent bullae along the striae distribution on her abdomen and extending to the flanks as demonstrated in Figure 1. The bullae were tender to touch and filled with clear fluid.

## 4 METHODS

Latest laboratory investigations showed typical features of nephrotic syndrome; hypoproteinemia (total protein 44 g/dL and albumin 5 g/dL) and abnormal lipid profile (total cholesterol 6.9 mmol/L, LDL 4.01 mmol/L and triglyceride 2.63 mmol/L). Additional laboratory findings included anemia (hemoglobin (Hb) 9.0 g/dL), neutrophilic leucocytosis (white blood cells 22.1 cells/mm^3^ and neutrophil 20.48 k/μL), lymphopenia (1.00 k/μL), thrombocytosis (PLT 541 × 10^9^/L), hypocalcaemia (Ca^2+^ 1.91 mmol/L), high microalbumin/creatinine ratio of 10380.9000 mg/g and abnormal urine analysis (152 RBC/HPF). Fluid aspiration culture and sensitivity was negative for the presence of bacteria and fungi, fluid albumin level was 0.3, and white blood cells count was 42 cells\mm^3^. Skin punch biopsy of one of the bullae with hematoxylin and eosin (H and E) stain revealed edematous dermis with relatively thinned horizontally oriented dermal collagen. Table 2 summarizes the abnormal laboratory results. These features supported the diagnosis of Bullous striae distensae. Patient was treated with 80 mg Furosemide once daily for 14 days and the bullae started to disappear slowly without any further intervention.

**TABLE 2:** Abnormal laboratory results.

Test/parameter	Result	Normal values
Complete blood count		
Hemoglobin	9.0 g/dL	Male:13.8–17.2 g/dL Female: 12.1–15.1 g/dL
White blood cells	22.1 cells/mm^3^	4500–11,000 cells/mm^3^
Neutrophil	20.48 k/μL	4.5–11.0 K/μL
Lymphocytes	1.00 k/μL	1.5–6.8 k/μL
Platelets	541 × 10^9^/L	150–400 × 10^9^/L
Lipid profile		
Cholesterol	6.9 mmol/L	<5.2 mmol/L
Low density lipoprotein	4.01 mmol/L	<2.6 mmol/L
Triglycerides	2.63 mmol/L	<1.7 mmol/L
Liver function test		
Total protein	44 g/dL	6.0–8.3 g/dL
Albumin	5 g/dL	3.4–5.4 g/dL
Fluid culture		
Fluid albumin	0.3 g/dL	3.5–5.5 g/dL
Chemistry		
Calcium	1.91 mmol/L	2.2–2.7 mmol/L
Urine analysis		
RBCs	152 RBC/HPF	<4 RBC/HPF
Kidney function tests		
Microalbumin/creatinine ratio	10380.9000 mg/g	<30 mg/g
Histopathology		
Punch biopsy of the lesions	Oedematous dermis with relatively thinned horizontally oriented dermal collagen	

## 5 CONCLUSION AND RESULTS

To conclude, the dramatic appearance of the fluid‐filled striae distensa may cause concerns among physicians who may not be familiar with this uncommon presentation, although they are benign. The emergence of bullous lesions in this context is likely attributed to the chronic use of systemic steroids and the underlying connective tissue weakening associated with the stria. Therefore, awareness of this rare complication and unusual clinical presentation is fundamental to prevent unnecessary investigations or treatments and ensure appropriate management of the underlying condition.

## 6 DISCUSSION

Striae distensae, are common skin lesions characterized by linear atrophic plaques directed perpendicularly to the skin.^2^, ^4^ They represent either, benign physiological changes as during pregnancy and adolescence, or a pathological sign of certain diseases.^4^ Rarely, bullae can develop within the striae.^5^, ^6^, ^7^ They exhibit lower hydration levels in both the epidermis and dermis, along with reduced dermal echo density and elasticity compared to normal skin.^10^ A rare variation of stria distensae, Bullous stria distensae, is characterized by the development of tense bullae within the stria. Almashat et al. suggested that patients with nephrotic syndrome and chronic steroid use may trigger bullous stria development, particularly in cases of anasarca.^2^, ^7^ The pathogenesis of BSD remains uncertain, however, it has been hypothesized that mechanical stress on the dermis leads to the rupture of the dermal vasculature and subsequent bullae formation. Seshadri et al. additionally proposed that the weak tensile strength of the stria may contribute to the build‐up of fluid within them. Besides, as the size of the stria increases, the likelihood of fluid accumulation also increases.^3^ Masoud et al. reported that patients on oral steroids, developing generalized body edema, tend to accumulate fluid, particularly in the striae. They further put forward a hypothesis that the phenomenon might result from the combined effect of both anasarca and steroid medication. Glucocorticoids were thought to enhance the collagen breakdown, reducing tensile strength allowing fluid to accumulate preferentially from anasarca within the stria spaces, forming fluid‐filled sacs.^5^

In our case, a 17‐year‐old female with nephrotic syndrome presented with an emergence of bullous lesions on her abdominal striae. The distinctive histopathologic characteristics of edematous dermis with a slightly thin and horizontally oriented dermal collagen confirmed the diagnosis of BSD. With regard to fluid aspiration culture and sensitivity report, the bullae were found to be sterile, which is consistent with a previous report by Seshadri et al.^3^ To the best of our knowledge, this is the fourth case reported the occurrence of bullous skin disease in a patient with nephrotic syndrome documented in the literature. The rapid weight gain and the presence of ascites in our patient may have resulted in increased mechanical stress on the dermis, which contributed to the development of BSD. The coexistence of lipodystrophy and hypoproteinemia in nephrotic syndrome may have further weakened dermal collagen, potentially increasing the patient's susceptibility to BSD development.^2^, ^4^ In our case and other reported cases, patients had hypoalbuminemia due to different pathologies and striae distensae. Four of the reported cases were associated with receiving high or fluctuating dose of systemic steroid for varying duration.^2^, ^3^, ^7^, ^9^ This phenomenon can be explained by the effect of decreased oncotic pressure on weakened or atrophic layers of skin.^6^ In this clinical context, differential diagnosis includes autoimmune bullous diseases, which can be ruled out through direct immunofluorescence. However, managing the underlying medical illness with diuresis can lead to spontaneous resolution, as puncture and aspiration of fluids alone would result in rapid fluid reaccumulation. Jogova et al. also highlighted that upon treatment with diuresis, flattening of the fluid‐filled stria were notable.^4^ Certainly, BSD management is primarily centred around addressing the underlying cause. In our case, the administration of diuretics to manage her ascites played a role in reducing the mechanical stress on the dermis, which resulted in a gradual resolution of the bullae.

The strength of our case report lies in its rarity, as we are only the second case report highlighting the potential advantage of employing diuresis as a management approach for BSD. It is crucial to attain optimal management of nephrotic syndrome to minimize the risk of BSD, considering the ongoing fluid shifts and the chronic use of corticosteroid. Several key clinical implications could inform future patient care and management strategies include: the necessity for clinicians to maintain a high index of suspicion for BSD in patients presenting with rapid‐onset bullous lesions, especially in the context of systemic diseases like nephrotic syndrome. The recognition of BSD as a differential diagnosis can prevent misdiagnosis and inappropriate treatment.

Future research is needed to enhance the understanding of the underlying pathogenesis of BSD. Additionally, further clinical trials are imperative to thoroughly assess the efficacy of diuresis as a viable management approach for BSD in order to establish the optimal management approaches for treating the condition. By shedding light on this barely explored area, the novelty of our findings contributes to enhancing the understanding of the potential therapeutic options for addressing bullous stria distensa.

## AUTHOR CONTRIBUTIONS

**Sumayyah I. Alrefaie:** Conceptualization; data curation; investigation; methodology; supervision; validation; visualization; writing – original draft; writing – review and editing. **Sarah B. Aljoudi:** Conceptualization; data curation; methodology; supervision; validation; visualization; writing – original draft; writing – review and editing. **Houriah Y. Nukaly:** Conceptualization; data curation; investigation; methodology; validation; visualization; writing – original draft; writing – review and editing. **Waseem AlHawasawi:** Conceptualization; data curation; methodology; validation; visualization; writing – original draft; writing – review and editing. **Jehad O. Hariri:** Conceptualization; data curation; methodology; project administration; supervision; validation; visualization; writing – original draft; writing – review and editing.

## FUNDING INFORMATION

None.

## CONFLICT OF INTEREST STATEMENT

The authors do not have any conflict of interest.

## CONSENT

Informed consent was obtained from the patient and/or legal guardian for the publication of this case report. The patient and/or legal guardian were informed that their identity would be kept confidential, and all personal identifying information would be removed to ensure anonymity. They were also made aware that the case report may be published in medical literature or presented at medical conferences. They were informed that their participation was voluntary and that they could withdraw their consent at any time.

## PHOTO CONSENT STATEMENT

Written consent for the use of photographs in this manuscript has been obtained from the individuals depicted in the images. The individuals have been assured of their anonymity and understand that these images will be used exclusively for educational and illustrative purposes within this manuscript.

## Data Availability

The data supporting this case report are available upon reasonable request. Interested parties may contact the corresponding author Sumayyah Alrefaie for access to the data related to this study.
